# 5,7-Dimethoxy­isobenzofuran-1(3*H*)-one

**DOI:** 10.1107/S1600536809031183

**Published:** 2009-08-15

**Authors:** Ming-Xue Sun, Xu Li, Wen-Yong Liu, Kai Xiao

**Affiliations:** a Laboratory of Toxicology & Pharmacology, Faculty of Naval Medicine, Second Military Medical University, Shanghai 200433, People’s Republic of China; b School of Traditional Chinese Materia Medica, Shenyang Pharmaceutical University, Shenyang 110016, People’s Republic of China

## Abstract

The asymmetric unit of the title compound, C_10_H_10_O_4_, which has been isolated from rhizoma Polygonum Cuspidatum, a Chinese folk medicine, contains two crystallographically independent mol­ecules. The mol­ecules are essentially planar, with a maximum deviation of 0.061 (2) Å from the best planes. The crystal packing is stabilized by weak inter­molecular C—H⋯O hydrogen-bonding inter­actions, with a stacking direction  of the mol­ecules parallel to [101].

## Related literature

For the synthesis of 5,7-dimethoxy­phthalide, see: Talapatra & Monoj (1980 ▶); Dang *et al.* (1999 ▶); Orito *et al.* (1995 ▶). For the title compound as an inter­mediate, see: Zuo *et al.* (2008 ▶); Lee *et al.* (2001 ▶). For the title compound as a by­product, see: Fürstner *et al.* (2000 ▶).
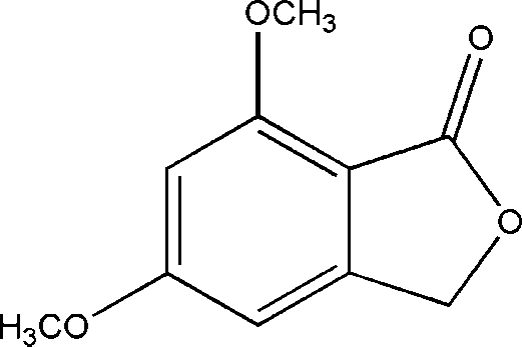


## Experimental

### 

#### Crystal data

C_10_H_10_O_4_
*M* *_r_* = 194.18
Monoclinic, 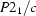

*a* = 8.532 (3) Å
*b* = 25.877 (10) Å
*c* = 8.374 (3) Å
β = 104.322 (6)°
*V* = 1791.5 (11) Å^3^
*Z* = 8
Mo *K*α radiation
μ = 0.11 mm^−1^
*T* = 293 K
0.12 × 0.12 × 0.10 mm

#### Data collection

Bruker SMART APEX CCD area-detector diffractometer
Absorption correction: multi-scan (*SADABS*; Sheldrick, 1996 ▶) *T*_min_ = 0.987, *T*_max_ = 0.989
7489 measured reflections
3216 independent reflections
1766 reflections with *I* > 2σ(*I*)
*R*_int_ = 0.062

#### Refinement

*R*[*F* ^2^ > 2σ(*F* ^2^)] = 0.052
*wR*(*F* ^2^) = 0.131
*S* = 0.93
3216 reflections
258 parameters
H-atom parameters constrained
Δρ_max_ = 0.18 e Å^−3^
Δρ_min_ = −0.19 e Å^−3^

### 

Data collection: *SMART* (Bruker, 2000 ▶); cell refinement: *SAINT* (Bruker, 2000 ▶); data reduction: *SAINT*; program(s) used to solve structure: *SHELXS97* (Sheldrick, 2008 ▶); program(s) used to refine structure: *SHELXL97* (Sheldrick, 2008 ▶); molecular graphics: *SHELXTL* (Sheldrick, 2008 ▶); software used to prepare material for publication: *SHELXTL* and *PLATON* (Spek, 2009 ▶).

## Supplementary Material

Crystal structure: contains datablocks I, global. DOI: 10.1107/S1600536809031183/wm2246sup1.cif

Structure factors: contains datablocks I. DOI: 10.1107/S1600536809031183/wm2246Isup2.hkl

Additional supplementary materials:  crystallographic information; 3D view; checkCIF report

## Figures and Tables

**Table 1:** Hydrogen-bond geometry (Å, °)

*D*—H⋯*A*	*D*—H	H⋯*A*	*D*⋯*A*	*D*—H⋯*A*
C6*A*—H6*A*⋯O1*B*^i^	0.93	2.51	3.397 (3)	161
C8*A*—H8*A*1⋯O2*B*^ii^	0.97	2.53	3.337 (3)	140
C6*B*—H6*B*⋯O1*A*^iii^	0.93	2.44	3.325 (3)	159

Symmetry codes: (i) 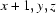; (ii) 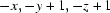; (iii) 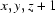.

**Fig. 1.**
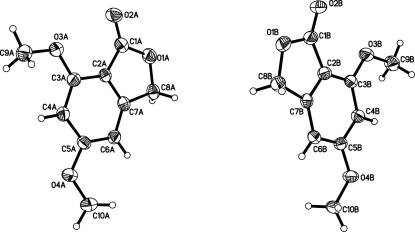
The molecular structure of 5,7-dimethoxyphthalide, showing the atom-labelling scheme. H atoms are shown as small spheres of arbitrary radius. Displacement ellipsoids are drawn at the 50% probability level.

**Fig. 2.**
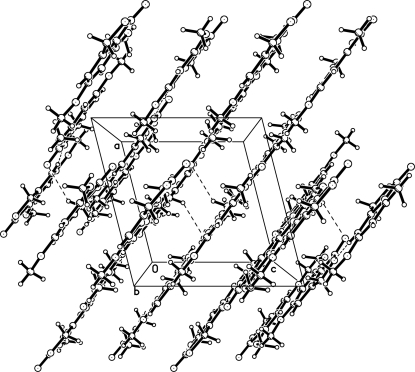
Molecular packing in the crystal, viewed along the b axis. Dashed lines indicate intermolecular C—H···O hydrogen bonds.

## supplementary crystallographic information

### Comment

The compound 5, 7-dimethoxyphthalide has been previously reported. It could be obtained by different synthetic strategies, e.g. from 5,7-dihydroxyphthalide (Talapatra & Monoj, 1980), 6-iodo-3-methoxybenzyl alcohols (Dang *et al.*, 1999) or 3,5-dimethoxybenzyl alcohol (Orito *et al.*,1995). It could act as an intermediate product in the process of synthesizing some significant compounds, such as 5,7-dimethoxy-4-methylphthalide and 5,7-dihydroxy-4-methylphthaIide (Zuo *et al.*, 2008), or mycophenolic acid and its analogs (Lee *et al.*, 2001). It was also reported as a byproduct in the synthesis of zearalenone and lasiodiplodin (Fürstner *et al.*, 2000). However, no structural details were provided. In this study, 5,7-dimethoxyphthalide was isolated from the rhizoma *Polygonum cuspidatum* as colorless prismatic crystals.

The molecule (Fig. 1 ) is essentially planar with a maximum deviation of 0.061 (2) Å from the best planes. The crystal packing is stabilized by weak intermolecular C—H···O hydrogen-bonding interactions with a stacking direction of the molecules parallel to [101] (Fig. 2 ).

### Experimental

The slices of the dried roots of *P. cuspidatum* (10 kg) were extracted with 60% aqueous acetone 3 times (24 h each) at room temperature. The solvent was evaporated in vacuo and some hydrophobic substances precipitated which were filtered off. The filtrate was concentrated to a suitable volume, then chromatographed on a Sephadex LH-20 column eluted with H_2_O, aqueous MeOH (10%-70%) and 50% acetone successively to give five fractions. The fraction eluated by 10% MeOH was subjected to MCI gel chromatography eluted with gradient aqueous MeOH solvent. The 30% aqueous MeOH eluate from the MCI column afforded the compound 5,7-dimethoxyphthalide as an amorphous powder. The powder was recrystallized in acetone and produced colourless prismatic crystals.

### Refinement

The H atoms were refined at calculated positions riding on the parent carbon atoms (C–H = 0.95–0.99 Å) with isotropic displacement parameters *U*_iso_(H) = 1.2U(Ceq) or 1.5U(–CH_3_). All CH_3_ hydrogen atoms were allowed to rotate but not to tip.

### Crystal data

**Table:**

C_10_H_10_O_4_	*F*(000) = 816
*M**_r_* = 194.18	*D*_x_ = 1.440 Mg m^−^^3^
Monoclinic, *P*2_1_/*c*	Mo *K*α radiation, λ = 0.71073 Å
Hall symbol: -P 2ybc	Cell parameters from 715 reflections
*a* = 8.532 (3) Å	θ = 2.6–21.3°
*b* = 25.877 (10) Å	µ = 0.11 mm^−^^1^
*c* = 8.374 (3) Å	*T* = 293 K
β = 104.322 (6)°	Prism, colourless
*V* = 1791.5 (11) Å^3^	0.12 × 0.12 × 0.10 mm
*Z* = 8	

### Data collection

**Table:**

Bruker SMART APEX CCD area-detector diffractometer	3216 independent reflections
Radiation source: fine-focus sealed tube	1766 reflections with *I* > 2σ(*I*)
graphite	*R*_int_ = 0.062
φ and ω scans	θ_max_ = 25.2°, θ_min_ = 1.6°
Absorption correction: multi-scan (*SADABS*; Sheldrick, 1996)	*h* = −7→10
*T*_min_ = 0.987, *T*_max_ = 0.989	*k* = −30→31
7489 measured reflections	*l* = −10→8

### Refinement

**Table:**

Refinement on *F*^2^	Secondary atom site location: difference Fourier map
Least-squares matrix: full	Hydrogen site location: inferred from neighbouring sites
*R*[*F*^2^ > 2σ(*F*^2^)] = 0.052	H-atom parameters constrained
*wR*(*F*^2^) = 0.131	*w* = 1/[σ^2^(*F*_o_^2^) + (0.053*P*)^2^] where *P* = (*F*_o_^2^ + 2*F*_c_^2^)/3
*S* = 0.93	(Δ/σ)_max_ < 0.001
3216 reflections	Δρ_max_ = 0.18 e Å^−^^3^
258 parameters	Δρ_min_ = −0.19 e Å^−^^3^
0 restraints	Extinction correction: *SHELXL97* (Sheldrick, 2008), Fc^*^=kFc[1+0.001xFc^2^λ^3^/sin(2θ)]^-1/4^
Primary atom site location: structure-invariant direct methods	Extinction coefficient: 0.0026 (5)

### Special details

**Table:**

Experimental. The powder of 5,7-dimethoxyphthalide was solved in acetone and produced colorless crystal.
Geometry. All e.s.d.'s (except the e.s.d. in the dihedral angle between two l.s. planes) are estimated using the full covariance matrix. The cell e.s.d.'s are taken into account individually in the estimation of e.s.d.'s in distances, angles and torsion angles; correlations between e.s.d.'s in cell parameters are only used when they are defined by crystal symmetry. An approximate (isotropic) treatment of cell e.s.d.'s is used for estimating e.s.d.'s involving l.s. planes.
Refinement. Refinement of *F*^2^ against ALL reflections. The weighted *R*-factor *wR* and goodness of fit *S* are based on *F*^2^, conventional *R*-factors *R* are based on *F*, with *F* set to zero for negative *F*^2^. The threshold expression of *F*^2^ > σ(*F*^2^) is used only for calculating *R*-factors(gt) *etc*. and is not relevant to the choice of reflections for refinement. *R*-factors based on *F*^2^ are statistically about twice as large as those based on *F*, and *R*- factors based on ALL data will be even larger.

### Fractional atomic coordinates and isotropic or equivalent isotropic displacement parameters (Å^2^)

**Table:**

	*x*	*y*	*z*	*U*_iso_*/*U*_eq_	
O1A	0.3975 (2)	0.42391 (7)	0.1615 (2)	0.0627 (6)	
O2A	0.2605 (3)	0.35976 (8)	0.0110 (2)	0.0674 (6)	
O3A	0.4002 (2)	0.25798 (7)	0.1709 (2)	0.0571 (6)	
O4A	0.8571 (2)	0.28610 (7)	0.6142 (2)	0.0628 (6)	
C1A	0.3670 (4)	0.37248 (11)	0.1270 (3)	0.0518 (8)	
C2A	0.4830 (3)	0.34270 (10)	0.2496 (3)	0.0417 (6)	
C3A	0.5069 (3)	0.28902 (10)	0.2724 (3)	0.0470 (7)	
C4A	0.6341 (3)	0.27237 (10)	0.3971 (3)	0.0471 (7)	
H4A	0.6529	0.2372	0.4141	0.057*	
C5A	0.7359 (3)	0.30831 (11)	0.4992 (3)	0.0487 (7)	
C6A	0.7112 (3)	0.36054 (10)	0.4795 (3)	0.0486 (7)	
H6A	0.7780	0.3842	0.5478	0.058*	
C7A	0.5835 (3)	0.37640 (10)	0.3545 (3)	0.0452 (7)	
C8A	0.5296 (3)	0.43027 (10)	0.3046 (3)	0.0542 (8)	
H8A1	0.4942	0.4478	0.3920	0.065*	
H8A2	0.6164	0.4500	0.2782	0.065*	
C9A	0.4125 (4)	0.20351 (11)	0.2069 (4)	0.0644 (9)	
H9A1	0.5177	0.1914	0.2025	0.097*	
H9A2	0.3312	0.1853	0.1270	0.097*	
H9A3	0.3968	0.1976	0.3150	0.097*	
C10A	0.9664 (4)	0.31963 (13)	0.7237 (4)	0.0764 (10)	
H10A	1.0202	0.3415	0.6614	0.115*	
H10B	1.0451	0.2994	0.7999	0.115*	
H10C	0.9072	0.3406	0.7833	0.115*	
O1B	−0.0885 (2)	0.46776 (7)	0.6553 (2)	0.0582 (5)	
O2B	−0.2380 (2)	0.53224 (8)	0.5190 (2)	0.0674 (6)	
O3B	−0.0891 (2)	0.63386 (7)	0.6714 (2)	0.0551 (5)	
O4B	0.3667 (2)	0.60524 (7)	1.1150 (2)	0.0539 (5)	
C1B	−0.1230 (3)	0.51938 (12)	0.6263 (3)	0.0522 (8)	
C2B	−0.0013 (3)	0.54864 (11)	0.7460 (3)	0.0454 (7)	
C3B	0.0172 (3)	0.60196 (10)	0.7725 (3)	0.0415 (7)	
C4B	0.1434 (3)	0.61849 (10)	0.8969 (3)	0.0440 (7)	
H4B	0.1590	0.6537	0.9162	0.053*	
C5B	0.2488 (3)	0.58354 (10)	0.9954 (3)	0.0420 (6)	
C6B	0.2297 (3)	0.53046 (10)	0.9714 (3)	0.0418 (6)	
H6B	0.2988	0.5069	1.0375	0.050*	
C7B	0.1032 (3)	0.51477 (9)	0.8449 (3)	0.0403 (6)	
C8B	0.0538 (3)	0.46110 (10)	0.7869 (3)	0.0522 (7)	
H8B1	0.1386	0.4442	0.7477	0.063*	
H8B2	0.0302	0.4406	0.8750	0.063*	
C9B	−0.0723 (4)	0.68778 (10)	0.7035 (4)	0.0620 (8)	
H9B1	−0.0863	0.6948	0.8117	0.093*	
H9B2	−0.1527	0.7062	0.6232	0.093*	
H9B3	0.0335	0.6988	0.6973	0.093*	
C10B	0.4778 (3)	0.57130 (11)	1.2222 (3)	0.0589 (8)	
H10D	0.4192	0.5485	1.2769	0.088*	
H10E	0.5531	0.5913	1.3028	0.088*	
H10F	0.5356	0.5514	1.1587	0.088*	

### Atomic displacement parameters (Å^2^)

**Table:**

	*U*^11^	*U*^22^	*U*^33^	*U*^12^	*U*^13^	*U*^23^
O1A	0.0734 (15)	0.0519 (13)	0.0597 (13)	0.0139 (11)	0.0105 (11)	0.0112 (10)
O2A	0.0663 (14)	0.0782 (15)	0.0517 (13)	0.0101 (12)	0.0029 (11)	0.0066 (11)
O3A	0.0585 (13)	0.0487 (13)	0.0580 (12)	0.0039 (10)	0.0028 (10)	−0.0025 (10)
O4A	0.0558 (12)	0.0596 (13)	0.0618 (13)	0.0065 (10)	−0.0066 (11)	−0.0011 (10)
C1A	0.055 (2)	0.059 (2)	0.0442 (18)	0.0101 (16)	0.0194 (16)	0.0078 (15)
C2A	0.0409 (16)	0.0434 (16)	0.0435 (16)	0.0030 (13)	0.0157 (13)	0.0015 (13)
C3A	0.0446 (17)	0.0492 (18)	0.0479 (18)	−0.0023 (14)	0.0129 (14)	−0.0027 (14)
C4A	0.0491 (17)	0.0413 (16)	0.0515 (17)	0.0026 (13)	0.0132 (15)	−0.0009 (13)
C5A	0.0420 (17)	0.0535 (19)	0.0498 (17)	0.0078 (14)	0.0095 (14)	0.0018 (14)
C6A	0.0468 (18)	0.0459 (17)	0.0530 (18)	−0.0022 (13)	0.0120 (15)	−0.0059 (13)
C7A	0.0444 (17)	0.0441 (17)	0.0520 (17)	0.0035 (13)	0.0215 (14)	0.0037 (14)
C8A	0.064 (2)	0.0491 (18)	0.0544 (18)	0.0077 (14)	0.0233 (16)	0.0054 (14)
C9A	0.069 (2)	0.0492 (19)	0.071 (2)	−0.0037 (15)	0.0088 (17)	−0.0008 (15)
C10A	0.068 (2)	0.078 (2)	0.069 (2)	0.0022 (18)	−0.0115 (18)	−0.0118 (18)
O1B	0.0522 (13)	0.0547 (13)	0.0659 (13)	−0.0078 (10)	0.0111 (10)	−0.0179 (10)
O2B	0.0443 (12)	0.0910 (16)	0.0590 (13)	−0.0001 (12)	−0.0020 (10)	−0.0168 (11)
O3B	0.0514 (12)	0.0544 (13)	0.0538 (12)	0.0078 (10)	0.0018 (9)	0.0017 (10)
O4B	0.0504 (12)	0.0478 (11)	0.0521 (12)	0.0024 (9)	−0.0089 (10)	−0.0010 (9)
C1B	0.0381 (18)	0.067 (2)	0.0520 (19)	−0.0063 (15)	0.0115 (15)	−0.0141 (15)
C2B	0.0382 (16)	0.0581 (18)	0.0409 (16)	−0.0021 (14)	0.0117 (13)	−0.0038 (14)
C3B	0.0381 (16)	0.0455 (17)	0.0410 (16)	0.0036 (13)	0.0098 (13)	0.0033 (13)
C4B	0.0428 (16)	0.0402 (16)	0.0474 (16)	−0.0020 (13)	0.0080 (14)	0.0000 (13)
C5B	0.0386 (16)	0.0485 (18)	0.0387 (15)	−0.0018 (13)	0.0090 (13)	−0.0029 (13)
C6B	0.0381 (16)	0.0438 (16)	0.0437 (16)	0.0047 (12)	0.0107 (13)	0.0030 (12)
C7B	0.0417 (16)	0.0397 (16)	0.0431 (15)	0.0003 (13)	0.0175 (13)	−0.0005 (13)
C8B	0.0486 (18)	0.0493 (18)	0.0581 (18)	−0.0027 (14)	0.0118 (14)	−0.0050 (14)
C9B	0.064 (2)	0.050 (2)	0.068 (2)	0.0100 (15)	0.0074 (16)	0.0063 (15)
C10B	0.0473 (19)	0.062 (2)	0.0578 (19)	0.0068 (15)	−0.0056 (15)	−0.0028 (15)

### Geometric parameters (Å, °)

**Table:**

O1A—C1A	1.373 (3)	O1B—C1B	1.376 (3)
O1A—C8A	1.437 (3)	O1B—C8B	1.434 (3)
O2A—C1A	1.200 (3)	O2B—C1B	1.202 (3)
O3A—C3A	1.346 (3)	O3B—C3B	1.357 (3)
O3A—C9A	1.440 (3)	O3B—C9B	1.422 (3)
O4A—C5A	1.355 (3)	O4B—C5B	1.354 (3)
O4A—C10A	1.428 (3)	O4B—C10B	1.434 (3)
C1A—C2A	1.458 (4)	C1B—C2B	1.463 (4)
C2A—C7A	1.377 (3)	C2B—C7B	1.372 (3)
C2A—C3A	1.410 (4)	C2B—C3B	1.400 (4)
C3A—C4A	1.376 (3)	C3B—C4B	1.368 (3)
C4A—C5A	1.408 (4)	C4B—C5B	1.393 (3)
C4A—H4A	0.9300	C4B—H4B	0.9300
C5A—C6A	1.371 (4)	C5B—C6B	1.392 (4)
C6A—C7A	1.374 (3)	C6B—C7B	1.373 (3)
C6A—H6A	0.9300	C6B—H6B	0.9300
C7A—C8A	1.495 (3)	C7B—C8B	1.497 (3)
C8A—H8A1	0.9700	C8B—H8B1	0.9700
C8A—H8A2	0.9700	C8B—H8B2	0.9700
C9A—H9A1	0.9599	C9B—H9B1	0.9599
C9A—H9A2	0.9599	C9B—H9B2	0.9599
C9A—H9A3	0.9599	C9B—H9B3	0.9599
C10A—H10A	0.9599	C10B—H10D	0.9599
C10A—H10B	0.9599	C10B—H10E	0.9599
C10A—H10C	0.9599	C10B—H10F	0.9599
			
C1A—O1A—C8A	110.8 (2)	C1B—O1B—C8B	110.8 (2)
C3A—O3A—C9A	116.7 (2)	C3B—O3B—C9B	117.3 (2)
C5A—O4A—C10A	117.5 (2)	C5B—O4B—C10B	117.7 (2)
O2A—C1A—O1A	120.2 (3)	O2B—C1B—O1B	120.0 (3)
O2A—C1A—C2A	132.1 (3)	O2B—C1B—C2B	132.7 (3)
O1A—C1A—C2A	107.7 (2)	O1B—C1B—C2B	107.3 (2)
C7A—C2A—C3A	119.4 (2)	C7B—C2B—C3B	120.2 (2)
C7A—C2A—C1A	108.8 (2)	C7B—C2B—C1B	109.1 (3)
C3A—C2A—C1A	131.8 (3)	C3B—C2B—C1B	130.6 (3)
O3A—C3A—C4A	125.1 (3)	O3B—C3B—C4B	124.3 (2)
O3A—C3A—C2A	116.7 (2)	O3B—C3B—C2B	118.0 (2)
C4A—C3A—C2A	118.2 (2)	C4B—C3B—C2B	117.7 (2)
C3A—C4A—C5A	120.4 (3)	C3B—C4B—C5B	121.3 (2)
C3A—C4A—H4A	119.8	C3B—C4B—H4B	119.4
C5A—C4A—H4A	119.8	C5B—C4B—H4B	119.4
O4A—C5A—C6A	124.8 (3)	O4B—C5B—C6B	123.7 (2)
O4A—C5A—C4A	113.5 (2)	O4B—C5B—C4B	114.9 (2)
C6A—C5A—C4A	121.6 (3)	C6B—C5B—C4B	121.3 (2)
C5A—C6A—C7A	117.1 (2)	C7B—C6B—C5B	116.4 (2)
C5A—C6A—H6A	121.5	C7B—C6B—H6B	121.8
C7A—C6A—H6A	121.5	C5B—C6B—H6B	121.8
C6A—C7A—C2A	123.3 (2)	C2B—C7B—C6B	123.1 (2)
C6A—C7A—C8A	128.5 (3)	C2B—C7B—C8B	107.9 (2)
C2A—C7A—C8A	108.2 (2)	C6B—C7B—C8B	129.0 (2)
O1A—C8A—C7A	104.5 (2)	O1B—C8B—C7B	104.8 (2)
O1A—C8A—H8A1	110.9	O1B—C8B—H8B1	110.8
C7A—C8A—H8A1	110.9	C7B—C8B—H8B1	110.8
O1A—C8A—H8A2	110.9	O1B—C8B—H8B2	110.8
C7A—C8A—H8A2	110.9	C7B—C8B—H8B2	110.8
H8A1—C8A—H8A2	108.9	H8B1—C8B—H8B2	108.9
O3A—C9A—H9A1	109.5	O3B—C9B—H9B1	109.5
O3A—C9A—H9A2	109.5	O3B—C9B—H9B2	109.5
H9A1—C9A—H9A2	109.5	H9B1—C9B—H9B2	109.5
O3A—C9A—H9A3	109.5	O3B—C9B—H9B3	109.5
H9A1—C9A—H9A3	109.5	H9B1—C9B—H9B3	109.5
H9A2—C9A—H9A3	109.5	H9B2—C9B—H9B3	109.5
O4A—C10A—H10A	109.5	O4B—C10B—H10D	109.5
O4A—C10A—H10B	109.5	O4B—C10B—H10E	109.5
H10A—C10A—H10B	109.5	H10D—C10B—H10E	109.5
O4A—C10A—H10C	109.5	O4B—C10B—H10F	109.5
H10A—C10A—H10C	109.5	H10D—C10B—H10F	109.5
H10B—C10A—H10C	109.5	H10E—C10B—H10F	109.5
			
C8A—O1A—C1A—O2A	−179.8 (2)	C8B—O1B—C1B—O2B	179.5 (2)
C8A—O1A—C1A—C2A	−0.5 (3)	C8B—O1B—C1B—C2B	−1.5 (3)
O2A—C1A—C2A—C7A	178.2 (3)	O2B—C1B—C2B—C7B	178.6 (3)
O1A—C1A—C2A—C7A	−1.0 (3)	O1B—C1B—C2B—C7B	−0.3 (3)
O2A—C1A—C2A—C3A	−0.6 (5)	O2B—C1B—C2B—C3B	0.5 (5)
O1A—C1A—C2A—C3A	−179.8 (3)	O1B—C1B—C2B—C3B	−178.5 (2)
C9A—O3A—C3A—C4A	6.2 (4)	C9B—O3B—C3B—C4B	−3.4 (4)
C9A—O3A—C3A—C2A	−172.6 (2)	C9B—O3B—C3B—C2B	177.8 (2)
C7A—C2A—C3A—O3A	177.0 (2)	C7B—C2B—C3B—O3B	180.0 (2)
C1A—C2A—C3A—O3A	−4.3 (4)	C1B—C2B—C3B—O3B	−2.0 (4)
C7A—C2A—C3A—C4A	−1.9 (4)	C7B—C2B—C3B—C4B	1.1 (4)
C1A—C2A—C3A—C4A	176.8 (3)	C1B—C2B—C3B—C4B	179.1 (2)
O3A—C3A—C4A—C5A	−178.2 (2)	O3B—C3B—C4B—C5B	−179.5 (2)
C2A—C3A—C4A—C5A	0.6 (4)	C2B—C3B—C4B—C5B	−0.7 (4)
C10A—O4A—C5A—C6A	0.5 (4)	C10B—O4B—C5B—C6B	0.2 (4)
C10A—O4A—C5A—C4A	−179.9 (2)	C10B—O4B—C5B—C4B	179.1 (2)
C3A—C4A—C5A—O4A	−178.9 (2)	C3B—C4B—C5B—O4B	−179.2 (2)
C3A—C4A—C5A—C6A	0.7 (4)	C3B—C4B—C5B—C6B	−0.2 (4)
O4A—C5A—C6A—C7A	179.0 (2)	O4B—C5B—C6B—C7B	179.6 (2)
C4A—C5A—C6A—C7A	−0.6 (4)	C4B—C5B—C6B—C7B	0.8 (4)
C5A—C6A—C7A—C2A	−0.8 (4)	C3B—C2B—C7B—C6B	−0.6 (4)
C5A—C6A—C7A—C8A	−179.5 (3)	C1B—C2B—C7B—C6B	−179.0 (2)
C3A—C2A—C7A—C6A	2.1 (4)	C3B—C2B—C7B—C8B	−179.8 (2)
C1A—C2A—C7A—C6A	−176.9 (2)	C1B—C2B—C7B—C8B	1.8 (3)
C3A—C2A—C7A—C8A	−178.9 (2)	C5B—C6B—C7B—C2B	−0.4 (4)
C1A—C2A—C7A—C8A	2.1 (3)	C5B—C6B—C7B—C8B	178.7 (2)
C1A—O1A—C8A—C7A	1.7 (3)	C1B—O1B—C8B—C7B	2.5 (3)
C6A—C7A—C8A—O1A	176.6 (2)	C2B—C7B—C8B—O1B	−2.6 (3)
C2A—C7A—C8A—O1A	−2.3 (3)	C6B—C7B—C8B—O1B	178.2 (2)

### Hydrogen-bond geometry (Å, °)

**Table:**

*D*—H···*A*	*D*—H	H···*A*	*D*···*A*	*D*—H···*A*
C6A—H6A···O1B^i^	0.93	2.51	3.397 (3)	161
C8A—H8A1···O2B^ii^	0.97	2.53	3.337 (3)	140
C6B—H6B···O1A^iii^	0.93	2.44	3.325 (3)	159

Symmetry codes: (i) *x*+1, *y*, *z*; (ii) −*x*, −*y*+1, −*z*+1; (iii) *x*, *y*, *z*+1.
